# Supplementary material for: Modeling glioblastoma heterogeneity as a dynamic network of cell states
Source: Mol Syst Biol. 2021 Sep 16;17(9):e10105. doi: 10.15252/msb.202010105 (PMC8444284; doi:10.15252/msb.202010105)
Supplement: Supplementary file 6 — Source Data for Figure 5 [file MSB-17-e10105-s004.zip › Figure5A_sourcedata/GSEA_3071/nosigdb_stateII.GseaPreranked.1622795151171/DOROSZKO_U3054MG_CLUSTER_4.html]

Details for gene set DOROSZKO\_U3054MG\_CLUSTER\_4[GSEA]

|  || Dataset | state23071 |
| Phenotype | NoPhenotypeAvailable |
| Upregulated in class | na\_pos |
| GeneSet | DOROSZKO\_U3054MG\_CLUSTER\_4 |
| Enrichment Score (ES) | 0.7127823 |
| Normalized Enrichment Score (NES) | 5.4583383 |
| Nominal p-value | 0.0 |
| FDR q-value | 0.0 |
| FWER p-Value | 0.0 |
Table: GSEA Results Summary

  

Fig 1: Enrichment plot: DOROSZKO\_U3054MG\_CLUSTER\_4      
 Profile of the Running ES Score & Positions of GeneSet Members on the Rank Ordered List

  

| PROBE | GENE SYMBOL | GENE\_TITLE | RANK IN GENE LIST | RANK METRIC SCORE | RUNNING ES | CORE ENRICHMENT || 1 | HIST1H1D |  |  | 0 | 2.323 | 0.0064 | Yes |
| 2 | CLSPN |  |  | 1 | 1.984 | 0.0119 | Yes |
| 3 | HIST1H4C |  |  | 2 | 1.902 | 0.0171 | Yes |
| 4 | RRM2 |  |  | 3 | 1.857 | 0.0223 | Yes |
| 5 | ANLN |  |  | 4 | 1.760 | 0.0271 | Yes |
| 6 | HIST1H1B |  |  | 7 | 1.669 | 0.0312 | Yes |
| 7 | TYMS |  |  | 8 | 1.665 | 0.0358 | Yes |
| 8 | MCM10 |  |  | 9 | 1.654 | 0.0403 | Yes |
| 9 | PCLAF |  |  | 10 | 1.638 | 0.0449 | Yes |
| 10 | CDC6 |  |  | 11 | 1.631 | 0.0494 | Yes |
| 11 | CDK1 |  |  | 14 | 1.540 | 0.0531 | Yes |
| 12 | DIAPH3 |  |  | 15 | 1.531 | 0.0573 | Yes |
| 13 | ZWINT |  |  | 16 | 1.510 | 0.0614 | Yes |
| 14 | HJURP |  |  | 17 | 1.490 | 0.0656 | Yes |
| 15 | ATAD5 |  |  | 18 | 1.469 | 0.0696 | Yes |
| 16 | FEN1 |  |  | 19 | 1.464 | 0.0737 | Yes |
| 17 | BRCA2 |  |  | 22 | 1.445 | 0.0771 | Yes |
| 18 | UBE2C |  |  | 23 | 1.438 | 0.0811 | Yes |
| 19 | CENPU |  |  | 24 | 1.416 | 0.0850 | Yes |
| 20 | RAD51AP1 |  |  | 25 | 1.384 | 0.0888 | Yes |
| 21 | CENPK |  |  | 26 | 1.382 | 0.0926 | Yes |
| 22 | CDCA4 |  |  | 27 | 1.376 | 0.0964 | Yes |
| 23 | MYBL2 |  |  | 28 | 1.362 | 0.1002 | Yes |
| 24 | FAM111B |  |  | 29 | 1.361 | 0.1039 | Yes |
| 25 | FBXO5 |  |  | 30 | 1.359 | 0.1077 | Yes |
| 26 | USP1 |  |  | 31 | 1.357 | 0.1114 | Yes |
| 27 | TCF19 |  |  | 32 | 1.331 | 0.1151 | Yes |
| 28 | DTL |  |  | 33 | 1.330 | 0.1188 | Yes |
| 29 | SLC43A3 |  |  | 34 | 1.323 | 0.1224 | Yes |
| 30 | PSMC3IP |  |  | 36 | 1.322 | 0.1258 | Yes |
| 31 | RRM1 |  |  | 37 | 1.310 | 0.1294 | Yes |
| 32 | ORC6 |  |  | 39 | 1.308 | 0.1327 | Yes |
| 33 | GINS2 |  |  | 40 | 1.306 | 0.1363 | Yes |
| 34 | CDCA5 |  |  | 41 | 1.289 | 0.1399 | Yes |
| 35 | TK1 |  |  | 42 | 1.281 | 0.1434 | Yes |
| 36 | AURKB |  |  | 44 | 1.276 | 0.1467 | Yes |
| 37 | MCM7 |  |  | 45 | 1.273 | 0.1502 | Yes |
| 38 | HELLS |  |  | 46 | 1.259 | 0.1537 | Yes |
| 39 | MCM8 |  |  | 48 | 1.253 | 0.1568 | Yes |
| 40 | BRCA1 |  |  | 49 | 1.249 | 0.1603 | Yes |
| 41 | CDT1 |  |  | 51 | 1.244 | 0.1635 | Yes |
| 42 | GINS1 |  |  | 52 | 1.239 | 0.1669 | Yes |
| 43 | CCNA2 |  |  | 53 | 1.230 | 0.1703 | Yes |
| 44 | DNAJC9 |  |  | 56 | 1.217 | 0.1731 | Yes |
| 45 | SKA3 |  |  | 57 | 1.215 | 0.1764 | Yes |
| 46 | PCNA |  |  | 58 | 1.214 | 0.1798 | Yes |
| 47 | CDC45 |  |  | 59 | 1.212 | 0.1831 | Yes |
| 48 | MAD2L1 |  |  | 61 | 1.199 | 0.1862 | Yes |
| 49 | UBE2T |  |  | 62 | 1.192 | 0.1895 | Yes |
| 50 | ATAD2 |  |  | 63 | 1.189 | 0.1927 | Yes |
| 51 | PRIM1 |  |  | 65 | 1.170 | 0.1957 | Yes |
| 52 | MSH2 |  |  | 66 | 1.163 | 0.1989 | Yes |
| 53 | RFC3 |  |  | 67 | 1.157 | 0.2021 | Yes |
| 54 | SPC25 |  |  | 68 | 1.156 | 0.2053 | Yes |
| 55 | MELK |  |  | 69 | 1.145 | 0.2084 | Yes |
| 56 | POLD3 |  |  | 70 | 1.143 | 0.2116 | Yes |
| 57 | WDHD1 |  |  | 71 | 1.133 | 0.2147 | Yes |
| 58 | TOP2A |  |  | 72 | 1.132 | 0.2178 | Yes |
| 59 | KIF2C |  |  | 73 | 1.132 | 0.2210 | Yes |
| 60 | MCM5 |  |  | 74 | 1.128 | 0.2241 | Yes |
| 61 | DSN1 |  |  | 75 | 1.122 | 0.2272 | Yes |
| 62 | CCNE2 |  |  | 76 | 1.121 | 0.2303 | Yes |
| 63 | IDH2 |  |  | 78 | 1.117 | 0.2331 | Yes |
| 64 | UHRF1 |  |  | 79 | 1.113 | 0.2362 | Yes |
| 65 | FAM111A |  |  | 80 | 1.112 | 0.2392 | Yes |
| 66 | MCM4 |  |  | 81 | 1.110 | 0.2423 | Yes |
| 67 | RBL1 |  |  | 82 | 1.110 | 0.2454 | Yes |
| 68 | GMNN |  |  | 83 | 1.108 | 0.2484 | Yes |
| 69 | RBBP8 |  |  | 86 | 1.100 | 0.2509 | Yes |
| 70 | CEP55 |  |  | 87 | 1.096 | 0.2539 | Yes |
| 71 | KIFC1 |  |  | 88 | 1.094 | 0.2569 | Yes |
| 72 | HIST1H1E |  |  | 90 | 1.088 | 0.2597 | Yes |
| 73 | XRCC2 |  |  | 92 | 1.076 | 0.2624 | Yes |
| 74 | VRK1 |  |  | 93 | 1.074 | 0.2653 | Yes |
| 75 | NDC80 |  |  | 94 | 1.074 | 0.2683 | Yes |
| 76 | POLA2 |  |  | 95 | 1.068 | 0.2712 | Yes |
| 77 | MASTL |  |  | 96 | 1.063 | 0.2742 | Yes |
| 78 | ERCC6L |  |  | 97 | 1.060 | 0.2771 | Yes |
| 79 | LRR1 |  |  | 98 | 1.060 | 0.2800 | Yes |
| 80 | TMPO |  |  | 99 | 1.056 | 0.2829 | Yes |
| 81 | NCAPG |  |  | 100 | 1.056 | 0.2859 | Yes |
| 82 | DHFR |  |  | 101 | 1.051 | 0.2888 | Yes |
| 83 | MYBL1 |  |  | 102 | 1.043 | 0.2916 | Yes |
| 84 | MND1 |  |  | 104 | 1.036 | 0.2942 | Yes |
| 85 | MCM3 |  |  | 105 | 1.035 | 0.2971 | Yes |
| 86 | MKI67 |  |  | 107 | 1.031 | 0.2996 | Yes |
| 87 | STIL |  |  | 108 | 1.031 | 0.3025 | Yes |
| 88 | FANCA |  |  | 109 | 1.029 | 0.3053 | Yes |
| 89 | BRIP1 |  |  | 110 | 1.029 | 0.3082 | Yes |
| 90 | TPX2 |  |  | 111 | 1.025 | 0.3110 | Yes |
| 91 | RFC2 |  |  | 112 | 1.022 | 0.3138 | Yes |
| 92 | CDCA8 |  |  | 113 | 1.018 | 0.3166 | Yes |
| 93 | TRIP13 |  |  | 114 | 1.014 | 0.3194 | Yes |
| 94 | KIF11 |  |  | 115 | 1.014 | 0.3222 | Yes |
| 95 | NCAPH |  |  | 116 | 1.013 | 0.3250 | Yes |
| 96 | NCAPD3 |  |  | 117 | 1.013 | 0.3278 | Yes |
| 97 | ASF1B |  |  | 120 | 1.007 | 0.3300 | Yes |
| 98 | SMC2 |  |  | 123 | 1.005 | 0.3323 | Yes |
| 99 | C12orf75 |  |  | 124 | 1.004 | 0.3350 | Yes |
| 100 | NUF2 |  |  | 125 | 1.004 | 0.3378 | Yes |
| 101 | DEPDC1 |  |  | 126 | 0.999 | 0.3406 | Yes |
| 102 | CHAF1A |  |  | 127 | 0.999 | 0.3433 | Yes |
| 103 | CKS1B |  |  | 128 | 0.999 | 0.3461 | Yes |
| 104 | TACC3 |  |  | 130 | 0.992 | 0.3485 | Yes |
| 105 | CENPL |  |  | 132 | 0.989 | 0.3510 | Yes |
| 106 | KIF20B |  |  | 133 | 0.989 | 0.3537 | Yes |
| 107 | MGME1 |  |  | 135 | 0.988 | 0.3562 | Yes |
| 108 | CDK4 |  |  | 137 | 0.986 | 0.3586 | Yes |
| 109 | FANCI |  |  | 138 | 0.984 | 0.3613 | Yes |
| 110 | WDR76 |  |  | 142 | 0.975 | 0.3632 | Yes |
| 111 | ESCO2 |  |  | 144 | 0.971 | 0.3656 | Yes |
| 112 | EXO1 |  |  | 145 | 0.969 | 0.3683 | Yes |
| 113 | PLP2 |  |  | 147 | 0.962 | 0.3707 | Yes |
| 114 | CKAP2L |  |  | 148 | 0.959 | 0.3733 | Yes |
| 115 | FANCB |  |  | 151 | 0.956 | 0.3754 | Yes |
| 116 | FIGNL1 |  |  | 152 | 0.954 | 0.3780 | Yes |
| 117 | E2F1 |  |  | 153 | 0.951 | 0.3806 | Yes |
| 118 | BLM |  |  | 156 | 0.942 | 0.3827 | Yes |
| 119 | NCAPG2 |  |  | 159 | 0.937 | 0.3847 | Yes |
| 120 | GGCT |  |  | 160 | 0.937 | 0.3873 | Yes |
| 121 | KIF23 |  |  | 161 | 0.936 | 0.3899 | Yes |
| 122 | CTPS1 |  |  | 162 | 0.934 | 0.3925 | Yes |
| 123 | DONSON |  |  | 163 | 0.933 | 0.3950 | Yes |
| 124 | SHCBP1 |  |  | 164 | 0.933 | 0.3976 | Yes |
| 125 | RTKN2 |  |  | 166 | 0.931 | 0.3999 | Yes |
| 126 | GTSE1 |  |  | 167 | 0.929 | 0.4025 | Yes |
| 127 | RPA2 |  |  | 169 | 0.929 | 0.4048 | Yes |
| 128 | CEP78 |  |  | 170 | 0.927 | 0.4073 | Yes |
| 129 | MTHFD1 |  |  | 173 | 0.914 | 0.4093 | Yes |
| 130 | BRI3BP |  |  | 175 | 0.909 | 0.4115 | Yes |
| 131 | MCM6 |  |  | 176 | 0.906 | 0.4140 | Yes |
| 132 | CENPM |  |  | 178 | 0.901 | 0.4162 | Yes |
| 133 | E2F7 |  |  | 179 | 0.901 | 0.4187 | Yes |
| 134 | SAC3D1 |  |  | 188 | 0.887 | 0.4189 | Yes |
| 135 | SPDL1 |  |  | 199 | 0.874 | 0.4186 | Yes |
| 136 | C21orf58 |  |  | 201 | 0.873 | 0.4207 | Yes |
| 137 | PLK4 |  |  | 205 | 0.870 | 0.4223 | Yes |
| 138 | NCAPH2 |  |  | 207 | 0.868 | 0.4244 | Yes |
| 139 | TFDP1 |  |  | 209 | 0.866 | 0.4265 | Yes |
| 140 | CSE1L |  |  | 210 | 0.866 | 0.4289 | Yes |
| 141 | PRIM2 |  |  | 212 | 0.865 | 0.4310 | Yes |
| 142 | PBK |  |  | 214 | 0.864 | 0.4331 | Yes |
| 143 | CEP152 |  |  | 217 | 0.860 | 0.4350 | Yes |
| 144 | C9orf40 |  |  | 218 | 0.860 | 0.4373 | Yes |
| 145 | CDCA3 |  |  | 219 | 0.858 | 0.4397 | Yes |
| 146 | CENPH |  |  | 221 | 0.855 | 0.4418 | Yes |
| 147 | RFC5 |  |  | 222 | 0.851 | 0.4441 | Yes |
| 148 | BUB1B |  |  | 223 | 0.851 | 0.4465 | Yes |
| 149 | CHAF1B |  |  | 229 | 0.845 | 0.4474 | Yes |
| 150 | CCDC15 |  |  | 230 | 0.845 | 0.4498 | Yes |
| 151 | ASPM |  |  | 231 | 0.842 | 0.4521 | Yes |
| 152 | ICMT |  |  | 232 | 0.842 | 0.4544 | Yes |
| 153 | EZH2 |  |  | 233 | 0.839 | 0.4567 | Yes |
| 154 | TCOF1 |  |  | 234 | 0.838 | 0.4590 | Yes |
| 155 | SLF1 |  |  | 237 | 0.834 | 0.4608 | Yes |
| 156 | HMGA1 |  |  | 238 | 0.834 | 0.4631 | Yes |
| 157 | MIS18A |  |  | 239 | 0.833 | 0.4654 | Yes |
| 158 | DBF4 |  |  | 240 | 0.832 | 0.4677 | Yes |
| 159 | RACGAP1 |  |  | 241 | 0.831 | 0.4700 | Yes |
| 160 | PRC1 |  |  | 242 | 0.830 | 0.4723 | Yes |
| 161 | TUBB6 |  |  | 244 | 0.828 | 0.4743 | Yes |
| 162 | C19orf48 |  |  | 248 | 0.826 | 0.4757 | Yes |
| 163 | KNTC1 |  |  | 249 | 0.825 | 0.4780 | Yes |
| 164 | NOP56 |  |  | 250 | 0.824 | 0.4803 | Yes |
| 165 | LMNB1 |  |  | 251 | 0.824 | 0.4825 | Yes |
| 166 | CHEK1 |  |  | 252 | 0.822 | 0.4848 | Yes |
| 167 | KIF18B |  |  | 254 | 0.815 | 0.4868 | Yes |
| 168 | NUP155 |  |  | 257 | 0.810 | 0.4885 | Yes |
| 169 | ATAD3A |  |  | 262 | 0.798 | 0.4896 | Yes |
| 170 | DNMT1 |  |  | 265 | 0.792 | 0.4912 | Yes |
| 171 | DKC1 |  |  | 268 | 0.789 | 0.4928 | Yes |
| 172 | YWHAH |  |  | 273 | 0.781 | 0.4939 | Yes |
| 173 | HMGB2 |  |  | 276 | 0.780 | 0.4955 | Yes |
| 174 | TUBA1C |  |  | 277 | 0.779 | 0.4976 | Yes |
| 175 | SMC4 |  |  | 279 | 0.777 | 0.4995 | Yes |
| 176 | DDIAS |  |  | 281 | 0.776 | 0.5013 | Yes |
| 177 | HSPB11 |  |  | 283 | 0.775 | 0.5032 | Yes |
| 178 | RFWD3 |  |  | 290 | 0.769 | 0.5037 | Yes |
| 179 | BARD1 |  |  | 295 | 0.760 | 0.5047 | Yes |
| 180 | POLE3 |  |  | 297 | 0.758 | 0.5065 | Yes |
| 181 | POLE |  |  | 305 | 0.750 | 0.5066 | Yes |
| 182 | MMS22L |  |  | 306 | 0.747 | 0.5087 | Yes |
| 183 | CENPO |  |  | 307 | 0.747 | 0.5107 | Yes |
| 184 | NSD2 |  |  | 308 | 0.747 | 0.5128 | Yes |
| 185 | KIF4A |  |  | 309 | 0.746 | 0.5148 | Yes |
| 186 | COPS3 |  |  | 310 | 0.746 | 0.5169 | Yes |
| 187 | CKS2 |  |  | 312 | 0.745 | 0.5187 | Yes |
| 188 | PRR11 |  |  | 314 | 0.743 | 0.5205 | Yes |
| 189 | ALYREF |  |  | 315 | 0.741 | 0.5225 | Yes |
| 190 | SKP2 |  |  | 317 | 0.740 | 0.5243 | Yes |
| 191 | SLBP |  |  | 318 | 0.740 | 0.5263 | Yes |
| 192 | CENPN |  |  | 320 | 0.737 | 0.5281 | Yes |
| 193 | PKMYT1 |  |  | 322 | 0.736 | 0.5298 | Yes |
| 194 | TIPIN |  |  | 326 | 0.732 | 0.5310 | Yes |
| 195 | NUP85 |  |  | 328 | 0.729 | 0.5327 | Yes |
| 196 | CENPW |  |  | 330 | 0.727 | 0.5345 | Yes |
| 197 | PHLDA2 |  |  | 332 | 0.726 | 0.5362 | Yes |
| 198 | WDR34 |  |  | 333 | 0.724 | 0.5382 | Yes |
| 199 | EBP |  |  | 336 | 0.721 | 0.5396 | Yes |
| 200 | MTHFD2 |  |  | 337 | 0.721 | 0.5416 | Yes |
| 201 | SPC24 |  |  | 340 | 0.719 | 0.5431 | Yes |
| 202 | LIG1 |  |  | 341 | 0.718 | 0.5450 | Yes |
| 203 | ORC1 |  |  | 342 | 0.717 | 0.5470 | Yes |
| 204 | RPA1 |  |  | 343 | 0.715 | 0.5490 | Yes |
| 205 | ARHGAP11A |  |  | 346 | 0.712 | 0.5504 | Yes |
| 206 | SPATA33 |  |  | 347 | 0.712 | 0.5524 | Yes |
| 207 | HPRT1 |  |  | 351 | 0.709 | 0.5535 | Yes |
| 208 | LMNB2 |  |  | 352 | 0.709 | 0.5555 | Yes |
| 209 | TOMM40 |  |  | 358 | 0.706 | 0.5560 | Yes |
| 210 | NT5C3A |  |  | 359 | 0.705 | 0.5580 | Yes |
| 211 | DTYMK |  |  | 364 | 0.701 | 0.5588 | Yes |
| 212 | POLE2 |  |  | 367 | 0.700 | 0.5602 | Yes |
| 213 | PSMG1 |  |  | 372 | 0.696 | 0.5610 | Yes |
| 214 | PRPF4 |  |  | 373 | 0.696 | 0.5629 | Yes |
| 215 | FKBP5 |  |  | 375 | 0.695 | 0.5645 | Yes |
| 216 | CENPJ |  |  | 376 | 0.695 | 0.5665 | Yes |
| 217 | TMEM106C |  |  | 377 | 0.694 | 0.5684 | Yes |
| 218 | KIF14 |  |  | 378 | 0.693 | 0.5703 | Yes |
| 219 | E2F8 |  |  | 379 | 0.691 | 0.5722 | Yes |
| 220 | PARPBP |  |  | 382 | 0.688 | 0.5735 | Yes |
| 221 | JPT2 |  |  | 384 | 0.687 | 0.5752 | Yes |
| 222 | RFC4 |  |  | 385 | 0.686 | 0.5771 | Yes |
| 223 | CCND3 |  |  | 390 | 0.683 | 0.5778 | Yes |
| 224 | MT2A |  |  | 393 | 0.682 | 0.5792 | Yes |
| 225 | SUPT16H |  |  | 394 | 0.682 | 0.5810 | Yes |
| 226 | EXOSC9 |  |  | 399 | 0.679 | 0.5818 | Yes |
| 227 | DIAPH1 |  |  | 400 | 0.678 | 0.5837 | Yes |
| 228 | TMEM97 |  |  | 403 | 0.676 | 0.5850 | Yes |
| 229 | KIF15 |  |  | 404 | 0.676 | 0.5869 | Yes |
| 230 | CTNNAL1 |  |  | 405 | 0.676 | 0.5887 | Yes |
| 231 | SMCHD1 |  |  | 406 | 0.676 | 0.5906 | Yes |
| 232 | ZNF367 |  |  | 408 | 0.673 | 0.5922 | Yes |
| 233 | BRIX1 |  |  | 410 | 0.672 | 0.5938 | Yes |
| 234 | CCDC34 |  |  | 411 | 0.672 | 0.5956 | Yes |
| 235 | TTF2 |  |  | 413 | 0.671 | 0.5972 | Yes |
| 236 | TEDC1 |  |  | 414 | 0.670 | 0.5990 | Yes |
| 237 | SGO2 |  |  | 415 | 0.670 | 0.6009 | Yes |
| 238 | GINS4 |  |  | 417 | 0.669 | 0.6025 | Yes |
| 239 | SAE1 |  |  | 418 | 0.669 | 0.6043 | Yes |
| 240 | KPNA2 |  |  | 420 | 0.669 | 0.6059 | Yes |
| 241 | FOXM1 |  |  | 423 | 0.667 | 0.6072 | Yes |
| 242 | SMC3 |  |  | 425 | 0.667 | 0.6087 | Yes |
| 243 | CENPS |  |  | 428 | 0.665 | 0.6100 | Yes |
| 244 | PHGDH |  |  | 429 | 0.664 | 0.6118 | Yes |
| 245 | CLN6 |  |  | 431 | 0.663 | 0.6134 | Yes |
| 246 | RMI1 |  |  | 433 | 0.660 | 0.6149 | Yes |
| 247 | ZWILCH |  |  | 436 | 0.659 | 0.6162 | Yes |
| 248 | LRRC59 |  |  | 437 | 0.658 | 0.6180 | Yes |
| 249 | NXT1 |  |  | 438 | 0.657 | 0.6198 | Yes |
| 250 | BUB3 |  |  | 441 | 0.657 | 0.6211 | Yes |
| 251 | POLA1 |  |  | 443 | 0.654 | 0.6226 | Yes |
| 252 | ACAT2 |  |  | 446 | 0.653 | 0.6239 | Yes |
| 253 | CCHCR1 |  |  | 450 | 0.651 | 0.6248 | Yes |
| 254 | PARP2 |  |  | 451 | 0.650 | 0.6266 | Yes |
| 255 | THOP1 |  |  | 455 | 0.648 | 0.6276 | Yes |
| 256 | RAD54L |  |  | 456 | 0.648 | 0.6294 | Yes |
| 257 | NCAPD2 |  |  | 457 | 0.647 | 0.6311 | Yes |
| 258 | NUDT15 |  |  | 461 | 0.646 | 0.6321 | Yes |
| 259 | MRE11 |  |  | 463 | 0.645 | 0.6336 | Yes |
| 260 | RAD18 |  |  | 465 | 0.642 | 0.6351 | Yes |
| 261 | ARPC5L |  |  | 471 | 0.640 | 0.6355 | Yes |
| 262 | HMMR |  |  | 475 | 0.639 | 0.6364 | Yes |
| 263 | DCTPP1 |  |  | 477 | 0.638 | 0.6379 | Yes |
| 264 | SSRP1 |  |  | 478 | 0.638 | 0.6397 | Yes |
| 265 | CKLF |  |  | 482 | 0.637 | 0.6406 | Yes |
| 266 | SHMT1 |  |  | 486 | 0.635 | 0.6415 | Yes |
| 267 | PGP |  |  | 492 | 0.631 | 0.6419 | Yes |
| 268 | FADS1 |  |  | 498 | 0.629 | 0.6422 | Yes |
| 269 | POP7 |  |  | 502 | 0.628 | 0.6431 | Yes |
| 270 | KNL1 |  |  | 504 | 0.628 | 0.6446 | Yes |
| 271 | BIRC5 |  |  | 505 | 0.628 | 0.6463 | Yes |
| 272 | ECT2 |  |  | 510 | 0.626 | 0.6469 | Yes |
| 273 | RRP7A |  |  | 515 | 0.621 | 0.6475 | Yes |
| 274 | GEN1 |  |  | 517 | 0.620 | 0.6490 | Yes |
| 275 | PANK2 |  |  | 519 | 0.618 | 0.6504 | Yes |
| 276 | HAUS8 |  |  | 527 | 0.613 | 0.6501 | Yes |
| 277 | SGO1 |  |  | 528 | 0.612 | 0.6518 | Yes |
| 278 | CDK2 |  |  | 530 | 0.612 | 0.6532 | Yes |
| 279 | ZGRF1 |  |  | 531 | 0.611 | 0.6549 | Yes |
| 280 | RPA3 |  |  | 532 | 0.611 | 0.6566 | Yes |
| 281 | C5orf34 |  |  | 533 | 0.611 | 0.6583 | Yes |
| 282 | UPF3B |  |  | 536 | 0.610 | 0.6594 | Yes |
| 283 | PPM1G |  |  | 539 | 0.609 | 0.6606 | Yes |
| 284 | BTG3 |  |  | 540 | 0.607 | 0.6622 | Yes |
| 285 | DDX39A |  |  | 542 | 0.605 | 0.6636 | Yes |
| 286 | RCC1 |  |  | 549 | 0.603 | 0.6636 | Yes |
| 287 | SPHK1 |  |  | 551 | 0.603 | 0.6650 | Yes |
| 288 | CENPX |  |  | 561 | 0.595 | 0.6642 | Yes |
| 289 | CENPQ |  |  | 569 | 0.590 | 0.6639 | Yes |
| 290 | CDC25A |  |  | 572 | 0.589 | 0.6649 | Yes |
| 291 | MIS18BP1 |  |  | 574 | 0.588 | 0.6663 | Yes |
| 292 | CENPF |  |  | 575 | 0.588 | 0.6679 | Yes |
| 293 | PCNT |  |  | 576 | 0.588 | 0.6695 | Yes |
| 294 | MAP3K20 |  |  | 587 | 0.583 | 0.6684 | Yes |
| 295 | TIMELESS |  |  | 591 | 0.583 | 0.6691 | Yes |
| 296 | NTM |  |  | 592 | 0.582 | 0.6708 | Yes |
| 297 | DDX11 |  |  | 596 | 0.579 | 0.6715 | Yes |
| 298 | EXOSC8 |  |  | 604 | 0.576 | 0.6712 | Yes |
| 299 | SMS |  |  | 608 | 0.575 | 0.6719 | Yes |
| 300 | SRPK1 |  |  | 610 | 0.574 | 0.6732 | Yes |
| 301 | MSH6 |  |  | 611 | 0.574 | 0.6748 | Yes |
| 302 | FBL |  |  | 612 | 0.573 | 0.6764 | Yes |
| 303 | CACYBP |  |  | 613 | 0.573 | 0.6780 | Yes |
| 304 | PPAT |  |  | 616 | 0.570 | 0.6790 | Yes |
| 305 | TUBG1 |  |  | 617 | 0.570 | 0.6806 | Yes |
| 306 | SMC1A |  |  | 618 | 0.570 | 0.6821 | Yes |
| 307 | CKAP2 |  |  | 619 | 0.569 | 0.6837 | Yes |
| 308 | CAD |  |  | 620 | 0.569 | 0.6853 | Yes |
| 309 | HAUS2 |  |  | 621 | 0.569 | 0.6869 | Yes |
| 310 | NUP188 |  |  | 622 | 0.569 | 0.6884 | Yes |
| 311 | FARSB |  |  | 625 | 0.568 | 0.6894 | Yes |
| 312 | RAD51D |  |  | 631 | 0.565 | 0.6896 | Yes |
| 313 | HAT1 |  |  | 632 | 0.565 | 0.6912 | Yes |
| 314 | SASS6 |  |  | 637 | 0.562 | 0.6916 | Yes |
| 315 | PPIF |  |  | 639 | 0.560 | 0.6929 | Yes |
| 316 | SPAG5 |  |  | 642 | 0.555 | 0.6939 | Yes |
| 317 | COMMD4 |  |  | 644 | 0.554 | 0.6951 | Yes |
| 318 | HAUS1 |  |  | 649 | 0.552 | 0.6955 | Yes |
| 319 | YEATS4 |  |  | 652 | 0.550 | 0.6965 | Yes |
| 320 | NUSAP1 |  |  | 654 | 0.550 | 0.6977 | Yes |
| 321 | DCK |  |  | 661 | 0.548 | 0.6976 | Yes |
| 322 | PRPS2 |  |  | 667 | 0.546 | 0.6977 | Yes |
| 323 | PAICS |  |  | 668 | 0.546 | 0.6992 | Yes |
| 324 | H2AFX |  |  | 671 | 0.544 | 0.7002 | Yes |
| 325 | MNS1 |  |  | 672 | 0.543 | 0.7017 | Yes |
| 326 | NUP107 |  |  | 675 | 0.539 | 0.7026 | Yes |
| 327 | MRPL17 |  |  | 682 | 0.537 | 0.7024 | Yes |
| 328 | WEE1 |  |  | 685 | 0.536 | 0.7033 | Yes |
| 329 | PPIH |  |  | 695 | 0.533 | 0.7023 | Yes |
| 330 | CIP2A |  |  | 696 | 0.533 | 0.7038 | Yes |
| 331 | FN3KRP |  |  | 704 | 0.531 | 0.7033 | Yes |
| 332 | NUP205 |  |  | 705 | 0.531 | 0.7048 | Yes |
| 333 | HADH |  |  | 706 | 0.530 | 0.7062 | Yes |
| 334 | PHTF2 |  |  | 707 | 0.530 | 0.7077 | Yes |
| 335 | EME1 |  |  | 726 | 0.525 | 0.7042 | Yes |
| 336 | POLD1 |  |  | 729 | 0.523 | 0.7051 | Yes |
| 337 | MCM2 |  |  | 734 | 0.521 | 0.7054 | Yes |
| 338 | CYB5A |  |  | 737 | 0.520 | 0.7063 | Yes |
| 339 | NETO2 |  |  | 740 | 0.519 | 0.7071 | Yes |
| 340 | NASP |  |  | 741 | 0.518 | 0.7086 | Yes |
| 341 | PHF19 |  |  | 745 | 0.518 | 0.7092 | Yes |
| 342 | UNG |  |  | 750 | 0.516 | 0.7095 | Yes |
| 343 | KDELC2 |  |  | 754 | 0.515 | 0.7101 | Yes |
| 344 | SLC1A5 |  |  | 757 | 0.514 | 0.7109 | Yes |
| 345 | SNX5 |  |  | 762 | 0.512 | 0.7112 | Yes |
| 346 | POLQ |  |  | 771 | 0.508 | 0.7104 | Yes |
| 347 | NUP62 |  |  | 777 | 0.505 | 0.7104 | Yes |
| 348 | CMC2 |  |  | 785 | 0.503 | 0.7099 | Yes |
| 349 | RAB5IF |  |  | 794 | 0.501 | 0.7091 | Yes |
| 350 | HACD3 |  |  | 800 | 0.498 | 0.7090 | Yes |
| 351 | EIF4EBP1 |  |  | 805 | 0.496 | 0.7093 | Yes |
| 352 | CPSF4 |  |  | 807 | 0.494 | 0.7104 | Yes |
| 353 | GART |  |  | 820 | 0.491 | 0.7084 | Yes |
| 354 | SRSF10 |  |  | 831 | 0.489 | 0.7070 | Yes |
| 355 | EEF1E1 |  |  | 840 | 0.488 | 0.7061 | Yes |
| 356 | ATIC |  |  | 863 | 0.481 | 0.7014 | Yes |
| 357 | SDC1 |  |  | 872 | 0.479 | 0.7005 | Yes |
| 358 | IKBIP |  |  | 876 | 0.477 | 0.7010 | Yes |
| 359 | ARL6IP6 |  |  | 880 | 0.476 | 0.7014 | Yes |
| 360 | CASP2 |  |  | 884 | 0.474 | 0.7019 | Yes |
| 361 | DERA |  |  | 886 | 0.474 | 0.7029 | Yes |
| 362 | NUP50 |  |  | 888 | 0.473 | 0.7040 | Yes |
| 363 | CISD2 |  |  | 891 | 0.473 | 0.7047 | Yes |
| 364 | CCDC138 |  |  | 903 | 0.470 | 0.7030 | Yes |
| 365 | SIGMAR1 |  |  | 924 | 0.465 | 0.6987 | Yes |
| 366 | RAD1 |  |  | 926 | 0.464 | 0.6997 | Yes |
| 367 | SPIN4 |  |  | 927 | 0.464 | 0.7010 | Yes |
| 368 | MRPL12 |  |  | 930 | 0.464 | 0.7017 | Yes |
| 369 | SVIP |  |  | 931 | 0.463 | 0.7030 | Yes |
| 370 | MCMBP |  |  | 934 | 0.462 | 0.7037 | Yes |
| 371 | ITGB3BP |  |  | 935 | 0.462 | 0.7050 | Yes |
| 372 | HIRIP3 |  |  | 936 | 0.462 | 0.7063 | Yes |
| 373 | C1orf35 |  |  | 937 | 0.462 | 0.7076 | Yes |
| 374 | SFI1 |  |  | 939 | 0.462 | 0.7085 | Yes |
| 375 | SRSF2 |  |  | 941 | 0.461 | 0.7095 | Yes |
| 376 | MORC4 |  |  | 946 | 0.460 | 0.7097 | Yes |
| 377 | GPN3 |  |  | 953 | 0.458 | 0.7093 | Yes |
| 378 | SNRPA1 |  |  | 954 | 0.458 | 0.7106 | Yes |
| 379 | BAZ1A |  |  | 960 | 0.455 | 0.7104 | Yes |
| 380 | RAD51C |  |  | 967 | 0.453 | 0.7100 | Yes |
| 381 | CDC7 |  |  | 971 | 0.451 | 0.7104 | Yes |
| 382 | KIF22 |  |  | 975 | 0.450 | 0.7109 | Yes |
| 383 | FANCD2 |  |  | 976 | 0.450 | 0.7121 | Yes |
| 384 | TEDC2 |  |  | 979 | 0.449 | 0.7128 | Yes |
| 385 | TRIM28 |  |  | 1004 | 0.444 | 0.7074 | No |
| 386 | USP37 |  |  | 1011 | 0.441 | 0.7069 | No |
| 387 | NR2C2AP |  |  | 1013 | 0.440 | 0.7078 | No |
| 388 | CASP8AP2 |  |  | 1025 | 0.436 | 0.7060 | No |
| 389 | ZNF519 |  |  | 1027 | 0.436 | 0.7069 | No |
| 390 | CCNE1 |  |  | 1045 | 0.429 | 0.7034 | No |
| 391 | FANCL |  |  | 1052 | 0.427 | 0.7029 | No |
| 392 | CEP57 |  |  | 1055 | 0.426 | 0.7035 | No |
| 393 | SLC25A40 |  |  | 1061 | 0.426 | 0.7033 | No |
| 394 | CHEK2 |  |  | 1073 | 0.424 | 0.7015 | No |
| 395 | NUDT21 |  |  | 1076 | 0.422 | 0.7021 | No |
| 396 | POC1A |  |  | 1080 | 0.421 | 0.7024 | No |
| 397 | SAMD1 |  |  | 1084 | 0.420 | 0.7027 | No |
| 398 | CTDSPL2 |  |  | 1109 | 0.415 | 0.6972 | No |
| 399 | SRRT |  |  | 1121 | 0.414 | 0.6953 | No |
| 400 | GMPS |  |  | 1126 | 0.413 | 0.6953 | No |
| 401 | UBA2 |  |  | 1131 | 0.412 | 0.6954 | No |
| 402 | CBX5 |  |  | 1132 | 0.412 | 0.6965 | No |
| 403 | POLR3K |  |  | 1140 | 0.410 | 0.6957 | No |
| 404 | CNTLN |  |  | 1142 | 0.409 | 0.6966 | No |
| 405 | BUB1 |  |  | 1145 | 0.408 | 0.6971 | No |
| 406 | SNRPF |  |  | 1150 | 0.407 | 0.6971 | No |
| 407 | TPRKB |  |  | 1168 | 0.403 | 0.6935 | No |
| 408 | DDB2 |  |  | 1189 | 0.399 | 0.6891 | No |
| 409 | CHTF18 |  |  | 1192 | 0.398 | 0.6896 | No |
| 410 | GPD2 |  |  | 1202 | 0.397 | 0.6882 | No |
| 411 | SNRPA |  |  | 1213 | 0.394 | 0.6866 | No |
| 412 | MZT1 |  |  | 1219 | 0.393 | 0.6863 | No |
| 413 | MRM2 |  |  | 1220 | 0.393 | 0.6873 | No |
| 414 | PHF5A |  |  | 1221 | 0.393 | 0.6884 | No |
| 415 | STIP1 |  |  | 1227 | 0.392 | 0.6881 | No |
| 416 | PSMC3 |  |  | 1241 | 0.389 | 0.6856 | No |
| 417 | ACD |  |  | 1246 | 0.387 | 0.6856 | No |
| 418 | TMEM38B |  |  | 1247 | 0.387 | 0.6866 | No |
| 419 | ZNF93 |  |  | 1265 | 0.384 | 0.6830 | No |
| 420 | RMI2 |  |  | 1279 | 0.381 | 0.6804 | No |
| 421 | POLE4 |  |  | 1281 | 0.381 | 0.6812 | No |
| 422 | CNOT9 |  |  | 1287 | 0.380 | 0.6809 | No |
| 423 | ABHD3 |  |  | 1288 | 0.380 | 0.6819 | No |
| 424 | MRPL14 |  |  | 1297 | 0.379 | 0.6807 | No |
| 425 | IFRD2 |  |  | 1308 | 0.377 | 0.6790 | No |
| 426 | HMGXB4 |  |  | 1322 | 0.375 | 0.6764 | No |
| 427 | EIF4A3 |  |  | 1332 | 0.372 | 0.6750 | No |
| 428 | MAGOHB |  |  | 1336 | 0.369 | 0.6752 | No |
| 429 | RNASEH2A |  |  | 1341 | 0.369 | 0.6751 | No |
| 430 | FDPS |  |  | 1348 | 0.367 | 0.6744 | No |
| 431 | TOPBP1 |  |  | 1355 | 0.366 | 0.6738 | No |
| 432 | SHMT2 |  |  | 1361 | 0.366 | 0.6734 | No |
| 433 | KLHL23 |  |  | 1369 | 0.362 | 0.6725 | No |
| 434 | ACP1 |  |  | 1396 | 0.358 | 0.6662 | No |
| 435 | SLC20A1 |  |  | 1400 | 0.357 | 0.6664 | No |
| 436 | POLR2D |  |  | 1414 | 0.353 | 0.6638 | No |
| 437 | AHCY |  |  | 1471 | 0.344 | 0.6492 | No |
| 438 | THOC3 |  |  | 1485 | 0.342 | 0.6466 | No |
| 439 | CCDC137 |  |  | 1494 | 0.342 | 0.6453 | No |
| 440 | DUSP23 |  |  | 1499 | 0.341 | 0.6451 | No |
| 441 | RABL6 |  |  | 1520 | 0.336 | 0.6405 | No |
| 442 | CBR3 |  |  | 1522 | 0.336 | 0.6411 | No |
| 443 | CBFB |  |  | 1548 | 0.332 | 0.6351 | No |
| 444 | NRM |  |  | 1550 | 0.331 | 0.6358 | No |
| 445 | TIMM10 |  |  | 1567 | 0.329 | 0.6322 | No |
| 446 | CENPP |  |  | 1569 | 0.329 | 0.6329 | No |
| 447 | TMEM237 |  |  | 1576 | 0.328 | 0.6321 | No |
| 448 | NPW |  |  | 1577 | 0.328 | 0.6330 | No |
| 449 | RFC1 |  |  | 1579 | 0.328 | 0.6337 | No |
| 450 | KHSRP |  |  | 1599 | 0.324 | 0.6293 | No |
| 451 | PRPS1 |  |  | 1620 | 0.320 | 0.6246 | No |
| 452 | PXMP2 |  |  | 1665 | 0.315 | 0.6133 | No |
| 453 | SLC2A4RG |  |  | 1672 | 0.314 | 0.6125 | No |
| 454 | LSM4 |  |  | 1676 | 0.313 | 0.6125 | No |
| 455 | GINS3 |  |  | 1694 | 0.312 | 0.6087 | No |
| 456 | VOPP1 |  |  | 1695 | 0.311 | 0.6096 | No |
| 457 | ANKRD9 |  |  | 1701 | 0.310 | 0.6090 | No |
| 458 | TMX1 |  |  | 1703 | 0.310 | 0.6096 | No |
| 459 | IPO9 |  |  | 1705 | 0.310 | 0.6102 | No |
| 460 | WDR54 |  |  | 1750 | 0.304 | 0.5988 | No |
| 461 | CD83 |  |  | 1838 | 0.294 | 0.5755 | No |
| 462 | GLO1 |  |  | 1867 | 0.290 | 0.5686 | No |
| 463 | PPP2R3B |  |  | 1870 | 0.290 | 0.5688 | No |
| 464 | ACOT7 |  |  | 1897 | 0.286 | 0.5624 | No |
| 465 | AEN |  |  | 1913 | 0.284 | 0.5590 | No |
| 466 | SDF2L1 |  |  | 1936 | 0.281 | 0.5537 | No |
| 467 | MRPL18 |  |  | 1972 | 0.276 | 0.5448 | No |
| 468 | CDC5L |  |  | 1986 | 0.274 | 0.5419 | No |
| 469 | PSMD1 |  |  | 1993 | 0.273 | 0.5410 | No |
| 470 | RBFA |  |  | 2020 | 0.270 | 0.5346 | No |
| 471 | PRADC1 |  |  | 2058 | 0.266 | 0.5251 | No |
| 472 | SKA2 |  |  | 2061 | 0.266 | 0.5252 | No |
| 473 | MPHOSPH9 |  |  | 2082 | 0.264 | 0.5204 | No |
| 474 | BLVRA |  |  | 2152 | 0.257 | 0.5020 | No |
| 475 | MXD3 |  |  | 2156 | 0.256 | 0.5019 | No |
| 476 | OXCT1 |  |  | 2185 | 0.253 | 0.4948 | No |
| 477 | CCDC167 |  |  | 2753 | -0.327 | 0.3386 | No |
| 478 | RSRC1 |  |  | 2966 | -0.367 | 0.2809 | No |
| 479 | PDLIM4 |  |  | 3220 | -0.433 | 0.2120 | No |
| 480 | ASRGL1 |  |  | 3362 | -0.483 | 0.1742 | No |
| 481 | OLFM1 |  |  | 3521 | -0.554 | 0.1320 | No |
| 482 | PPP1R14C |  |  | 3537 | -0.564 | 0.1294 | No |
| 483 | HUNK |  |  | 3569 | -0.582 | 0.1224 | No |
| 484 | CDCA7L |  |  | 3642 | -0.637 | 0.1042 | No |
| 485 | MCUB |  |  | 3790 | -0.778 | 0.0656 | No |
| 486 | TCEAL7 |  |  | 3819 | -0.808 | 0.0601 | No |
| 487 | FBLN1 |  |  | 3909 | -0.965 | 0.0381 | No |
| 488 | HES6 |  |  | 3961 | -1.092 | 0.0270 | No |
| 489 | DRAXIN |  |  | 4018 | -1.374 | 0.0153 | No |
| 490 | PMP2 |  |  | 4082 | -2.398 | 0.0044 | No |
Table: GSEA details [plain text format]

  

Fig 2: DOROSZKO\_U3054MG\_CLUSTER\_4: Random ES distribution      
 Gene set null distribution of ES for **DOROSZKO\_U3054MG\_CLUSTER\_4**
